# Editorial: MicroRNA Signaling

**DOI:** 10.3389/fcell.2020.612425

**Published:** 2020-10-30

**Authors:** Lijun Wang, Carmen Elena Condrat, Saumya Das, Junjie Xiao, Dragos Creţoiu

**Affiliations:** ^1^Cardiac Regeneration and Ageing Lab, Institute of Cardiovascular Sciences, School of Life Science, Shanghai University, Shanghai, China; ^2^School of Medicine, Shanghai University, Shanghai, China; ^3^Alessandrescu-Rusescu National Institute of Mother and Child Health, Fetal Medicine Excellence Research Center, Bucharest, Romania; ^4^Cardiovascular Division of the Massachusetts General Hospital and Harvard Medical School, Boston, MA, United States; ^5^Department of Cell and Molecular Biology and Histology, Carol Davila University of Medicine and Pharmacy, Bucharest, Romania

**Keywords:** microRNA, signaling, disease, circulating microRNA, hypertrophy

MicroRNAs (miRNAs, miRs) are a class of non-coding small RNA molecules composed of 21–25 nucleotides. Recent studies have shown that miRNAs play important regulatory roles in various physiological and pathological processes. MicroRNAs (notably present in biofluids) also have value as biomarkers of disease, and can be therapeutic targets in certain diseases. This Research Topic entitled “MicroRNA Signaling” highlights the disease-related molecular pathways where miRNAs play an important role. The 21 selected papers in this special Research Topic cover the role of miRNAs in both physiological and pathological processes, including embryonic development, cancers, cardiovascular diseases, neurological disorders. Additionally, they discuss the emerging role of circulating miRNAs as potential biomarkers with a possible role as novel mediators of intercellular communication and gene expression regulation.

Research papers from this topic were all directed toward a better understanding of miRNA regulation of signal transduction networks in various diseases and incorporated a wide range of methods. Altered expression of miRNAs and their function as a major contributor of various cellular pathways in cancers have been widely implicated. In this topic, Neagu et al. provided an outstanding overview about the miRNAs in the diagnosis and prognosis of skin cancer, as well as putative future therapeutic targets. In addition, the main miRNA detection technologies that are used to evaluate miRNAs in tissues and body fluids were also summarized, along with their advantages and limitations. Xia et al. explored the role of miR-486 in pancreatic cancer. They found that miR-486 promoted proliferation and cell cycle progression of Capan-2 pancreatic cancer cells via targeting PTEN and inhibition of miR-486 was proposed as a novel therapy for pancreatic cancer. Interestingly, Wang H. et al. investigated the mechanism of SHU00238, which is an isoxazole derivative, and has been reported to inhibit colorectal tumor growth. They found that SHU00238 promotes colorectal cancer cell apoptosis through regulating miR-4701-3p and miR-4793-3p, and provides a potential drug target and therapeutic strategy for colorectal cancer. Also, Wang X. et al. demonstrated that lncRNA LINC00319/miR-3127/RAP2A plays an important role in cell growth and invasion of bladder cancer. Thus, enhancing the expression of miR-3127 or inhibition of LINC00319 might represent a promising therapeutic strategy for bladder cancer treatment. In addition, Yusof et al. provided a comprehensive dataset of human miRNAs and (PIWI)-interacting RNAs expression derived from next-generation sequencing in papillary thyroid carcinoma. In addition, the utility of extracellular vesicles derived miR-21-5p and miR-92a-3p for hepatocellular carcinoma diagnosis was explored by Sorop et al.

Five original research papers and one review in this topic focusing on the role of miRNAs involved in regulating the cardiovascular system. Zhang et al. gave an outstanding review of the regulatory effects of non-coding RNAs in exercise-mediated cardiac protection, highlighting the regulation of miRNAs in adaptive cardiovascular changes after exercise and discussing future perspectives. Autophagy is required for exercise-induced beneficial adaptive changes. Qi et al. explored the mechanism of autophagy in exercise-induced physiological cardiac hypertrophy and found that miR-26b-5p, miR-204-5p, and miR-497-3p were involved in modulating physiological cardiac hypertrophy by targeting their respective autophagy genes. Interestingly, Zhu et al. reported that a circular RNA circNFIB attenuated cardiac fibrosis via sponging miR-433, and the circNFIB–miR-433 axis might represent a novel therapeutic approach for the treatment of fibrotic diseases. Cheng et al. demonstrated that inhibition of long non-coding RNA H19 protects the endothelium against high glucose-induced inflammation and oxidative stress by means of regulation of the H19/miR-29b/VEGFA signaling pathways. Tesovnik et al. explored the function of extracellular vehicles (EVs) derived human miRNAs in type 1 diabetes, describing the importance of EVs-derived miRNAs in the regulation of the immune system and treatment of type 1 diabetes. Finally, Zhou et al. profiled the circulating miRNAs in response to cardiopulmonary exercise testing and acute exercise training in healthy adults and found changes of serum circulating miRNAs miR-20a and miR-21.

MiRNAs contribution to other physiological and pathological processes was also explored in this topic. Lin et al. studied the embryo-secreted miR-10b in bovine embryos and found that miR-10b promotes embryonic cells apoptosis via HOXA1. Zhu et al. sequenced the small RNAs at the epiboly stage in MZdgcr8 mutant embryos and investigated the role of Dgcr8 in miRNA processing. The contribution of miR-216 in bovine primary muscle cells proliferation and differentiation, and the elderly plasma circulating miRNAs regulation in response to sarcopenia were examined by Yang et al. and He et al., respectively. Gao et al. studied neurogenic differentiation from amniotic epithelial cells and found that lncRNA Maternally Expressed 3 (MEG3) could act as a competing endogenous RNA of miR-128-3p that activated the Notch signaling pathway to increase neuronal differentiation.  Ma et al. identified Agrin participates in regulating BoNT/A-induced nerve sprouting via miR-144-agrin-Musk signaling, and possibly be a therapeutic target for neurological disorders. Also, Han et al. explored the mechanism that contributed to the dysregulation of miR-384 in T-helper cell 17 (Th17) polarization. They found that the signal transducer and activator of transcription 3 (STAT3) activated miR-384/SOCS3 axes to regulate Th17 polarization. Pang et al. identified and analyzed the miRNA and mRNA expression profiles that were involved in agonistic behavior in Chinese Mitten Crab via a deep sequence method. They found that miRNAs and mRNAs mainly focused on the RAS, cAMP, AMPK, and energy metabolism pathways after a fight. Finally, Barbu et al. gave an outstanding overview about miRNAs that are involved in interacting with various signaling pathways during viral infections, providing insight into further investigations and potential clinical applications.

In conclusion, this Research Topic provided detailed regulatory roles and molecular mechanisms for understanding miRNAs signaling pathways in physiological and pathological processes. The number and range of papers considered within the subject of the microRNA signaling demonstrate the complexity of the topic. The papers comprising this Research Topic greatly contributed to further understanding of the regulatory signaling pathway and potential applications for the diagnosis of miRNAs.

## Author Contributions

LW drafted the manuscript. CC, SD, JX, and DC significantly contributed to the drafts toward the final product and critically reviewed the manuscript. All authors listed have read this editorial and approved it for publication.

## Conflict of Interest

The authors declare that the research was conducted in the absence of any commercial or financial relationships that could be construed as a potential conflict of interest.

